# Automatic Swimming Activity Recognition and Lap Time Assessment Based on a Single IMU: A Deep Learning Approach

**DOI:** 10.3390/s22155786

**Published:** 2022-08-03

**Authors:** Erwan Delhaye, Antoine Bouvet, Guillaume Nicolas, João Paulo Vilas-Boas, Benoît Bideau, Nicolas Bideau

**Affiliations:** 1 M2S Laboratory (Movement, Sports & Health), University Rennes 2, ENS Rennes, 35170 Bruz, France; antoine.bouvet@ens-rennes.fr (A.B.); guillaume.nicolas@univ-rennes2.fr (G.N.); benoit.bideau@univ-rennes2.fr (B.B.); nicolas.bideau@univ-rennes2.fr (N.B.); 2 MIMETIC-Analysis-Synthesis Approach for Virtual Human Simulation, INRIA Rennes Bretagne Atlantique, Campus de Beaulieu, 263 Av. Général Leclerc, 35042 Rennes, France; 3 LABIOMEP Laboratory (Porto Biomechanics Laboratory), Faculty of Sport, CIFI2D, University of Porto, 4200-450 Porto, Portugal; jpvb@fade.up.pt

**Keywords:** swimming monitoring, inertial measurement units, deep learning, human activity recognition, lap time

## Abstract

This study presents a deep learning model devoted to the analysis of swimming using a single Inertial Measurement Unit (IMU) attached to the sacrum. Gyroscope and accelerometer data were collected from 35 swimmers with various expertise levels during a protocol including the four swimming techniques. The proposed methodology took high inter- and intra-swimmer variability into account and was set up for the purpose of predicting eight swimming classes (the four swimming techniques, rest, wallpush, underwater, and turns) at four swimming velocities ranging from low to maximal. The overall F1-score of classification reached 0.96 with a temporal precision of 0.02 s. Lap times were directly computed from the classifier thanks to a high temporal precision and validated against a video gold standard. The mean absolute percentage error (MAPE) for this model against the video was 1.15%, 1%, and 4.07%, respectively, for starting lap times, middle lap times, and ending lap times. This model is a first step toward a powerful training assistant able to analyze swimmers with various levels of expertise in the context of in situ training monitoring.

## 1. Introduction

There is a growing trend in swimming, as in many other sports, to monitor human physiological function, technical skills, and performance during in situ training. Indeed, current swimming training programs make monitoring of the swimmer’s training load and performance a key concept [1]. To do this, several performance devices and sensors are becoming more readily available for athletes and allow performance to be quantified more precisely [2,3]. Among conventional devices, global positioning systems cannot be used during indoor swimming while cameras suffer from optical occlusions and data processing that is non-automatic as well as time consuming, making reliable assessment hard to achieve. To overcome these limitations, inertial measurement units (IMU) have become a relevant solution for monitoring and performance analysis [4]. Moreover, they do not require any external equipment, are not confined to restricted capture areas, and have the ability to continuously monitor swimmers in a real environment over a prolonged period without huge technical and logistical pressure [5]. While IMUs are now widely used for human movement analysis in various fields, such as clinical, ergonomics, and sports [2], swimming possesses features that make it distinctive in comparison to other sports. Indeed, swimming is characterized by the existence of four strokes that include large 3D sculling movements along with different intra-cycle phases (glide, catch, pull, recovery) and transitional phases (the start, turn, and underwater phases). Moreover, the design of a swimming training session is carried out on the basis of work and recovery times and exercise intensities that impact the biomechanics of swimming [6,7]. This observation provides meaning and is an opportunity to provide valuable indicators (e.g., lap and rest times, stroke count and frequency, time spent underwater) in the frame of a monitoring process. Another feature lies in the medium in which the swimmer moves, which may influence raw IMU data. Indeed, gyroscopes are highly sensitive to temperature variation [8,9,10], which may engender significant drift, particularly for temperatures higher than 20 °C [11]. Another consideration is due to the oscillations between body and device, i.e., soft tissue artefacts (STA) [12], which compete with drift as the most crucial source of error. As STAs are both motor task- and subject-dependent [13], it can be assumed that these oscillations in water differ from those recorded on land, despite fixation techniques that may limit the oscillations. These considerations (biomechanical features and specificity of the medium in which the swimmer moves) clearly illustrate the requirements for the development of a specific method dedicated to the analysis of swimming activities based on IMUs. Several valuable pieces of information can be extracted from IMU data during swimming phases, as well as during the underwater and turn phases [4,5,14]: (1) temporal parameters, e.g., lap and rest times; (2) instantaneous kinematic parameters, e.g., intracyclic variations; and (3) stroke characteristics, e.g., stroke count and frequency and time spent underwater. In a monitoring context, the identification of at least all swimming phases (butterfly, backstroke, breaststroke, frontcrawl) and transitional phases (start, turn, and underwater phases) along with lap and rest times is required. This can be achieved through the development of a human activity recognition (HAR) methodology.

In this regard, various IMU-based classification algorithms have been proposed in swimming [15,16,17,18,19,20]. Most published swimming activity recognition methods select features from a set of engineered metrics by relying on conventional mathematical operations in the time and frequency domains [21]. These methods include detection of extrema and/or zero-crossing and filtering techniques applied to the signals based on a set of predefined thresholds. Using an accelerometer located on the sacrum, Refs. [17,22] proposed a threshold-based methodology from a combination of orientation and component energy to determine the stroke type with 96.1% accuracy. This method, sometimes combined with a gyroscopic signal, has been widely used in the literature for stroke recognition [23,24,25]; as well as for the wall push, turn, and touch identification sometimes used for lap time (LT) calculation [19,25,26,27,28,29,30,31]. LT calculation refers to identified peaks in the acceleration signal. However, this threshold-based technique can lead to mistakes, as false positive peaks such as those induced by a powerful leg kick may be confused with the beginning or end of a lap. Despite their ease of use, these methods suffer from several drawbacks: (1) it is necessary to find an optimal parameterisation for the processing functions in order to set the population under study; (2) it is relatively difficult to deal with the inter- and intravariability of IMU signals due to the differences in athletes’ technique; and (3) it has difficulty handling environments with interference from other swimmers. These factors compromise the ability of threshold-based techniques to provide automatic robust classification without generalisation issues.

Recently, thanks to improvements in computing capabilities, many studies have used machine learning (ML) approaches that can automatically classify sequences of features or directly learn features from the signal in order to increase the accuracy of general human activity recognition [2]. More specifically, for swimming activity classification these approaches include Support Vector Machine (SVM) [32], Random Forest (RF) [33], and Principal Component Analysis (PCA) [15] approaches. These methods have been mainly used for feature selection in order to train a classifier for swimming style classification. Such methods are often designed from large time windows for feature extraction, resulting in poor temporal precision that may not be suited for monitoring elite swimming. Thus, one recent paper compared sliding window techniques to an intra-stroke segmentation technique and pointed out the possibility of performing stroke-by-stroke analysis [20]. Moreover, depending on the objectives with which the model is developed, the cross-validation technique has to be justified. When working with an identified group such as a swimming team, it can be of great interest to develop a specific model for the team in order to make the classification more informative and precise. In this case, either (1) a holdout or (2) k-fold cross validation can be sufficient [34]. However, if the aim is to develop a model that can be generalized to wide population panels, it is essential to separate the training, validation, and testing sets with different subjects in order to assess the generalization power of the model using a subject-independent cross-validation technique. For HAR, two main methodologies of cross-validation are detailed in the literature: (1) leave-one-out cross-validation, which typically has a high computational cost; and (2) hold-one-out cross-validation, which is generally used when the algorithm requires considerable computation to iterate, as with deep learning (DL) models [21].

The last approach takes advantages of recent enhancements in Deep Learning (DL). Indeed, such techniques have made it possible to achieve promising accuracy, reproducibility, and temporal precision in exhaustive human activity recognition. DL refers mainly to neural networks that exploit many layers of nonlinear transformation processing for non-human-dependant feature extraction and classification. They are organised hierarchically, with each layer processing the outputs of the previous layer. DL for time series classification relies on the ability to automate the critical feature extraction module via learning from signals, using, for example, layers in Convolutional Neural Networks (CNN) or in Long Short-Term Memory (LSTM). Previous studies using IMU data have reported that using DL is likely to surpass conventional ML algorithms in HAR [35]. More specifically, in Swinmming Activity Recognition (SAR), CNN-based methods, which can automatically extract discriminative features with convolutional kernels, have demonstrated better and more generalizable performances than conventional methods using predesigned features such as descriptive statistics [16]. An interesting DL approach for human swimming style recognition and lap counting has been developed in which a convolutional neural network (CNN) with high performance was successfully used in swimming style recognition. Furthermore, studies based on recurrent neural networks (RNN) such as LSTM have demonstrated interesting results for SAR [36] with a Bi-LSTM (Bidirectional Long Short-Term Memory) network. Data records were collected from 40 swimmers and were labeled into eight classes: Unknown, Null, Freestyle, Breaststroke, Backstroke, Butterfly, Turn, and Kick. The Bi-LSTM method was able to perform activity classification with an average F1 score of 91.39%. To attain such performance, the network used pre-extracted statistical features as inputs instead of direct IMU signals. However, LSTM and Bi-LSTM are designed to process and make predictions from available sequences of data. In contrast, CNN is designed to exploit “spatial correlations” in data, making them perform well when identifying shapes from images [37]. Therefore, it would be interesting to train a bi-LSTM-based model on swimming without pre-extracted features, as in [36] except with raw IMU data, in order to input data to be temporal series. All of the above-mentioned studies relied on publicly available [16] databases with a non-video-based labelling of activity into five classes: the four swimming techniques and a transition phase including the rest of the swimmer’s activity (turn and break). Despite valuable contributions, this approach requires the development of new classes to provide more widely applicable insights for monitoring in real training conditions. From this perspective, it is be necessary to integrate other essential variables of training control, such as LT and a classification separating the underwater, turn, and rest phases. To the best of our knowledge, ML and DL have never been used to compute LT automatically. Finally, while most of these models are well suited to considering homogeneous data, they show large performance drops in ecological conditions or when applied on high level populations, for example. Indeed, many papers developed their models with a homogeneous population [25] and/or at homogeneous paces [15]. Therefore, it may be important to gather a database in which the participants have heterogeneous levels of swimming (inducing inter-subject variability) and swim at heterogeneous paces during their training (inducing intra-subject variability) in order to ensure that the model can be widely generalized.

This study is primarily aimed at developing a deep learning model devoted to the analysis of swimming using a single IMU attached to the sacrum. It should be able to classify swimming activities at the different velocities that may occur during a full training session. Moreover, the proposed methodology should be generalizable to a wide panel of swimmers through the use of a database with high inter- and intra-swimmer variability. Secondary purposes are to compute LT directly from the classifier with high temporal precision and to validate LT values using a video gold standard.

## 2. Materials and Methods

Data were gathered from several sessions of experiments including 35 swimmers. Participants were 11 females and 24 males (age: 23.23 ± 8.85 y.o.; height: 176.48 ± 9.61 cm; mass: 65.81 ± 10.79 kg; BMI: 21.05 ± 2.46 m·kg^−2^; swim experience: 8.88 ± 2.95 y.o.) with a swimming level of recreational to second league level. All participants signed an informed consent form in agreement with the French Ethical Committee (approval obtained under reference 2021-A00250-41) and conducted in accordance with the 1975 Declaration of Helsinki.

### 2.1. Experimental Set-Up

The experiment took place in an indoor 25 m pool. The participants were instrumented with one waterproofed IMU (Cometa WaveTrack, Milano, Italy) composed of a 3D accelerometer and 3D gyroscope. Accelerometer and gyroscope data were sampled at the same frequency of 280 Hz using a full scale set at ±8 g and ±1000deg· s^−1^, respectively.

The sensor was placed on the sacrum at the middle point between the two posterior superior iliac spines, then fixed with double-sided tape and secured with waterproof medical adhesive (Tegaderm, 3M, Cergy-Pontoise, France). The IMU described a coordinate system defined with *x*-axis pointing cranially, *y*-axis pointing laterally, and *z*-axis pointing posteriorly.

Three cameras sampled at 30 Hz were used to identify lap events and swimming activities, and served as a reference for validation purposes. Two cameras (GoPro Hero 8, San Mateo, CA, USA) were placed under the waterline at one meter from each poolside in the direction of the wall in order to record the side-view of the pool and corresponding swimmer activity (turn, touch, wall-push). The third camera (Handycam HDR-XR550, Sony, Minato-ku, Tokyo, Japan) was used as a travelling camera above the water. The three cameras were synchronised with the IMU using the flashlight of the LED embedded in the IMU sensor. This procedure was repeated at the beginning and the end of each measurement in order to ensure perfect synchronisation over the whole swimming trial.

Moreover, each LT was recorded by an operator with a stopwatch in order to compare IMU lap times (LT IMU) with the stopwatch (LT MAN) and assess their validity with regard to the cameras, considered as the true label, obtained by post hoc labeling based on the video footage (LT CAM).

### 2.2. Experimental Protocol

**Figure 1:**
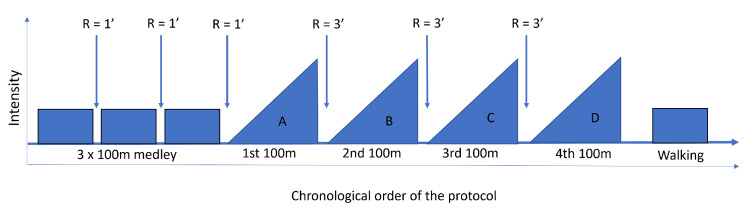
Overview of the protocol.

After a standardized warm-up, participants attended one measurement session divided into two exercises. First, swimmers were asked to perform a set of 3 × 100 m medleys, with 1 min of passive rest between each, swum in the conventional order (butterfly, backstroke, breaststroke, front crawl) at a moderate pace. After 3 min of rest, they randomly performed one 100 m per swimming style with 3 min of passive rest between each. Swimmers were asked to increase their velocity across each 100 m in order to record different swim speeds, with the intention of inducing larger intra-subject variability for model training. Thus, the order used by the swimmers was to first swim the first 25 m at low speed, then the second 25 m at moderate speed, the third at high speed, and the last 25 m at maximum speed. Finally, swimmers walked 50 m along the pool in order to train the classifier not to misclassify a swimmer walking along the poolside as a swimming phase. The resulting data set was therefore representative of a large variety of strokes, non-swimming phases, and paces from swimmers with various levels of expertise. The overall protocol is depicted in Figure 1. Moreover, each LT was recorded by an operator with a stopwatch.

### 2.3. Ground Truth Activity Video Labelling and Lap Time Assessment

From the entire dataset, eight phases defining swimming activities were defined. These phases and their defined starting and ending occurrences are summarised in Table 1 and were used to extract timecodes from the video gold standard. Then, the IMU signal was labelled with this subsequent activity. Based on this digitization of swim activity, video-based lap times (LT CAM) were computed according to specific definitions depending on the swimming technique and the lap type, namely, the beginning, middle, and last lap of an interval, respectively denoted as LT START, LT MIDDLE, and LT END (see Table 3).

### 2.4. IMU Data Processing and Deep Learning Model

#### 2.4.1. IMU Data Preprocessing

**Figure 2:**
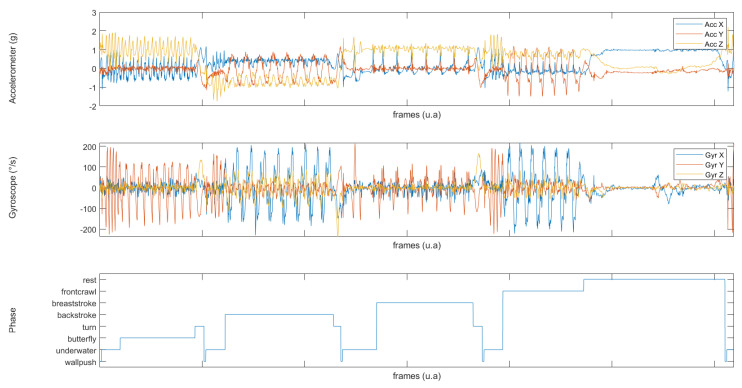
Correspondance between the signal and the true activity of the swimmer: (**top**,**center**) accelerometer and gyroscope signals for all axes, and (**bottom**) true activity of the signal, presented as activity labelling.

Let X be the raw data that correspond to the sensor’s output time series:

$$X=\left[\begin{array}{c} x_{1},x_{2},\ldots ,x_{t} \end{array}\right]$$

where $x_{i}$ denotes the accelerometer and gyroscope values at time *t*. Raw data from the IMU sensor were filtered using a second order Butterworth low-pass filter with a 10 Hz cut-off frequency and downsampled to 50 Hz in order to lower the computational cost. An example is shown in Figure 2. Standardization was applied to the input data. The mean (${\bar{X}}_{ij}$) and the standard deviation (σ) were computed for each IMU channel. Then standardization was applied to each time series following Equation (1):

(1) $$X_{ij}^{norm}=\frac{X_{ij}-{\bar{X}}_{ij}}{\sigma }$$

where $X_{ij}$ is a given IMU channel, ${\bar{X}}_{ij}$ is the mean of the corresponding IMU channel accross all subjects, and σ is the corresponding standard deviation. The time series data were then transformed in a preprocessed time series, $X^{\prime }$:

$$X^{\prime }=\left[\begin{array}{c} x_{1}^{\prime },x_{2}^{\prime },\ldots ,x_{n}^{\prime } \end{array}\right]$$

where *n* is the number of total dimensions. This preprocessing is needed in order to preserve the signal characteristics, including relevant information about the activity.

Following data recording and preprocessing steps, a two step methodology was developed. The first step was performed using a deep learning model trained from the database. The second step filtered the raw predictions of the DL model in order to eliminate prediction mistakes through a previously engineered procedure [21].

#### 2.4.2. Segmentation

The data segmentation step identified the segments of the preprocessed data most likely to contain information about activities. Thus, each data segment $s_{i}=\left[\begin{array}{c} t_{s},t_{e} \end{array}\right]$ was defined by its start time $t_{s}$ and end time $t_{e}$ within the preprocessed time series. Finally, the segmentation step output a set of segments S containing a potential activity

$$S=\left[\begin{array}{c} s_{1},s_{2},\ldots ,s_{n} \end{array}\right]$$

In the present paper, segmentation was performed using a sliding window procedure. The window size mainly affects the delay in the recognition process. As the optimal window size is not obvious a priori, it can influence recognition performance [38]. The window size usually corresponds to a tradeoff between segmentation precision and computational cost. This size is dependant on the type and structure of the underlying time series data. A window of 90 frames, i.e., 1.8 s duration, was selected in order to integrate at least one period of each phase (swimming techniques, turn, wallpush, etcetera).

The training set was then shuffled in order to prevent overfitting. The X input of the network to be trained was a n×m×f 3D matrix, where *n* is the number of windows, m=6 is the number of IMU channels, and f=90 is the window length.

#### 2.4.3. Network Architecture

Let Y be the predefined activity, i.e, the labels that correspond to the activity performed by the swimmer at the median frame of the time window

$$\left\{Y_{1},Y_{2},\ldots ,Y_{m}\right\}$$

where m=8 denotes the number of activity types. These activity types are defined in Table 1 and are the outputs of the model.

Let F be the model that predicts an activity sequence $\hat{Y}$ based on preprocessed sensor data $X^{\prime }$:

$$\hat{Y}=\left\{{\hat{Y}}_{1},\ldots ,{\hat{Y}}_{n}\right\}=F\left(X^{\prime }\right),{\hat{Y}}_{i}\in Y$$

Let Y∗ be the true activity sequence (ground truth):

$$Y*=\left\{Y{*}_{1},\ldots ,Y{*}_{n}\right\},{Y*}_{i}\in Y$$

where *n* corresponds to the length of the sequence (n≥m). The objective of swimming activity recognition is thus to learn the model by minimizing the discrepancy between the predicted activity $\hat{Y}$ and the ground truth activity Y∗.

A DL model was considered in order to predict the swimming activity at each time step. The architecture is summarized in Table 2. The network was implemented using Tensorflow [39] and Keras [40] and relied on LSTM cells [41] used bidirectionnaly [42].

Learning lasted ten epochs, an epoch being the number of passes through the entire training dataset the DL algorithm has completed. The initial learning rate was fixed to 0.001 after performing a grid search optimisation on a range of learning rates from 0.1 to 0.00001, as this value led to the smallest loss for ten epochs. This selected learning rate was scheduled with an epoch-dependant decline during training, as follows:

(2) $$\alpha =\frac{0.001}{800^{\frac{epochs}{100}}}$$

Finally, based on testing set raw prediction sequence $\hat{Y}$, an algorithm developed with Matlab (The Mathworks™, R2020b, Natick, MA, USA) was applied in order to remove artefacts and prediction skips by filtering the activity predictions [21].

Parameter weight initialization of each layer followed Xavier uniform initialization [43]. An ADAM optimizer updated parameters weights during training [44]. The last computation layer of the network prior to the classification output layer was composed of a multilayer perceptron (MLP). Batch normalization was applied to this layer in order to facilitate training and reduce internal covariate shift in this deeper layer [45]. Finally, the loss function is a sparse categorical entropy one, as the present model corresponds to a multi-class classification problem.

#### 2.4.4. Training and Testing Sets

Model training was based on $X^{\prime }$ time series, which were used to construct the different sets (training, validation, and testing). A holdout-subject cross-validation (HOSCV) consisting of extracting the validation set and testing set with subject-wise data, was employed. One subject was randomly chosen to form these sets, and all other subjects accounted for the training set. While this validation procedure relies on the same principle as holdout cross-validation, it illustrates how the model works when tested on a subject that is not part of the database, making the results more generalizable [21].

We recorded 990 laps during the experiments, along with calculations of LT CAM during each lap. However, due to material bugs and human mistakes during the execution of the protocol, only 952 laps and 870 laps were retained to compute LT IMU and LT MAN, respectively. The method used to describe LT CAM and LT MAN is sumarized in Table 3. LT IMUs were computed using $\hat{Y}$ and based on class transitions, as shown in Table 4.

**Table 3:** Definition of phase and events to compute LT CAM and to monitor swimmer activity with stopwatch (LT MAN).

	START	MIDDLE	END
Frontcrawl and Backstroke	Time difference between the beginning of initial wall push and next wall push	Time difference between a wall push and next wall push	Time difference between last wall push and final touch with the hand
Butterfly and Breaststroke	Time difference between initial wall push and the first simultaneous touch with the hand	Time difference between a touch with the hand on the wall and the next touch on the wall	Time difference between the last touch on the wall with the hand and the final touch with hand

#### 2.4.5. Model Performance Analysis

Evaluation of the model’s performance in predicting swimmer activity consisted of applying the model on the validation and testing sets, respectively. Thus, the performance of the network was monitored during training by its accuracy on the validation set. After training, the ability of the model to be generalized was evaluated using the performance obtained on the testing set. The predictions of swimming activities provided by the model were compared with the ground truth, and the performance of the model was assessed by: (1) precision (i.e., the number of true positives over the sum of true positives and false positives); (2) recall (i.e., the number of true positives over the number of true positives plus the number of false negatives); and (3) an F1-score (i.e., precision time recall over precision plus recall) confusion matrix.

Evaluation of the model;s performance in predicting the lap times was conducted through a statistical analysis using R Studio (Version 1.2.5033, RStudio, Inc., Boston, MA, USA). The agreement between LT IMU and LT CAM and between LT IMU and LT MAN was determined using: (1) the bias and its 95% confidence interval; (2) the Typical Error of Measurement (TEM) [46] and its 95% confidence interval; (3) a Bland–Altman plot and analysis [47,48]; and (4) the Mean Absolute Percentage Error (MAPE) and its standard deviation, additionally expressed in seconds as an indicator of the expected measurement error [49]. Moreover, in order to assess the accuracy of the model as a function of lap type (i.e., starting lap, intermediary lap, and ending lap), the IMU-based, camera-based and stopwatch-based lap time agreement was calculated according to category (LT START, LT MIDDLE, and LT END).

## 3. Results

### 3.1. Performance of Swim Activity Recognition

The results presented here are based on the predictions made by the model for each iteration of the sliding window. Based on the video labeling, the model is supposed to predict the performed activity at the median frame of the sliding window, which in this work is the 45th frame of each window. They are presented in Table 5.

Prior to the filtering step, the average overall precision on the testing set was 0.77 and the weighted average precision was 0.92. As the dataset was unbalanced, assigning weights to the classes as a function of their number of samples tended to improve the precision, as the model trained better on those classes. Short classes such as wallpush, turns, and underwater had lower precision, respectively, at 0.17, 0.58, and 0.71. However, activities that were more well represented in the dataset (strokes and rest) were predicted more precisely, as 0.98, 0.96, 0.83, 0.96, and 0.99, respectively, for the butterfly, backstroke, breaststroke, front crawl, and rest.

This tendency remains the same for recall, with lower values for short classes and higher values for stroke styles and rest. Indeed, recall was 0.08, 0.71, and 0.71 for the wallpush, turns, and, underwater phase, respectively, and 0.83, 0.94, 0.95, 0.96, and 0.99, respectively, for the butterfly, backstroke, breaststroke, front crawl, and rest.

The combination of those last two metrics, that is, the F1-score, was 0.91 on the testing set. F1 scores per phase were between 0.11 and 0.99 for wallpush and rest, respectively. For the four swimming techniques, F1 scores were between 0.89 and 0.96, with 0.90, 0.89, 0.95, and 0.96, respectively, for the butterfly, breaststroke, backstroke, and front crawl. For the non-swimming phases, the underwater and turn phases had F1 scores of 0.91 and 0.82, respectively.

The second step of the model, filtering, removed artefacts due to misclassifications ${\hat{Y}}_{f}$. Whatever the class, performance metrics were systematically improved after filtering. The average overall precision on the testing set increased to 0.88 and, the weighted average precision increased to 0.96. Short classes such as wallpush, turns, and underwater had lower precision, at 0.53, 0.75, and 0.92, respectively. Strokes and rest reached 0.99, 0.98, 0.89, 0.99, and 0.99, respectively, for the butterfly, backstroke, breaststroke, front crawl, and rest.

This hierarchy remained the same for recall, with lower values for short classes and higher values for stroke styles and rest. Recall values were 0.19, 0.91, and 0.91 for the wallpush, turns, and underwater phase, respectively, and 0.88, 0.99, 0.98, 0.99, and 0.99 for the butterfly, backstroke, breaststroke, front crawl, and rest, respectively.

Finally, F1-score reached an overall value of 0.96. However, results showed heterogeneity in the performance repartition. Indeed, the wallpush F1-score was 0.28, while the rest F1-score was 0.99. For the four swimming techniques the F1-score was between 0.93 and 0.99, at 0.93, 0.94, 0.99, and 0.99, respectively, for the butterfly, breaststroke, backstroke, and front crawl. Regarding non-swimming phases, the underwater and turn phases reached an F1-score of 0.91 and 0.82, respectively.

### 3.2. Lap Time Assessment

The agreement between the lap times obtained from the IMU and the lap times obtained from the gold standard video are presented in Table 6.

**Figure 3:**
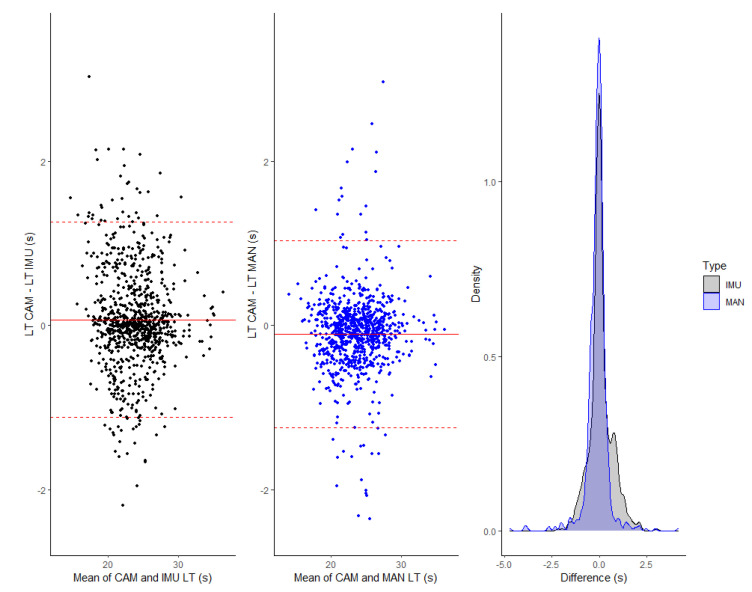
(**Left**) Bland–Altman plot of IMU and gold standard video, (**middle**) Bland–Altman plot of stopwatch and gold standard video, and (**right**) density distribution of difference between lap times assessed by IMU and gold standard video and by stopwatch and video.

LT IMUs are slightly overestimated, with an error (systematic ± random) of 0.06 s (−0.05; 0.14) ± 0.60 s (0.57; 0.63) and MAPE of 1.77 ± 1.82%, corresponding to 0.42 s ± 0.43. Figure 3 presents the Bland–Altman plot and density distribution of the differences between LT IMU and LT CAM, summarized in Table 6. Reported errors are mainly around ±1 s.

The agreement between the lap times obtained from the stopwatch and those obtained from gold standard video are presented in Table 7.

LT MAN are slightly underestimated, with an error (systematic ± random) of −0.10 s (−0.14; 0.07) ± 0.58 s (0.55; 0.61) and MAPE of 1.43 ± 2.04%, corresponding to 0.34 s ± 0.48 s. Figure 3 presents the Bland–Altman plot and density distribution of the differences between LT MAN and LT CAM, summarized in Table 7. Reported errors are mainly around ±0.75 s.

#### Lap Time Type Analysis

**Figure 4:**
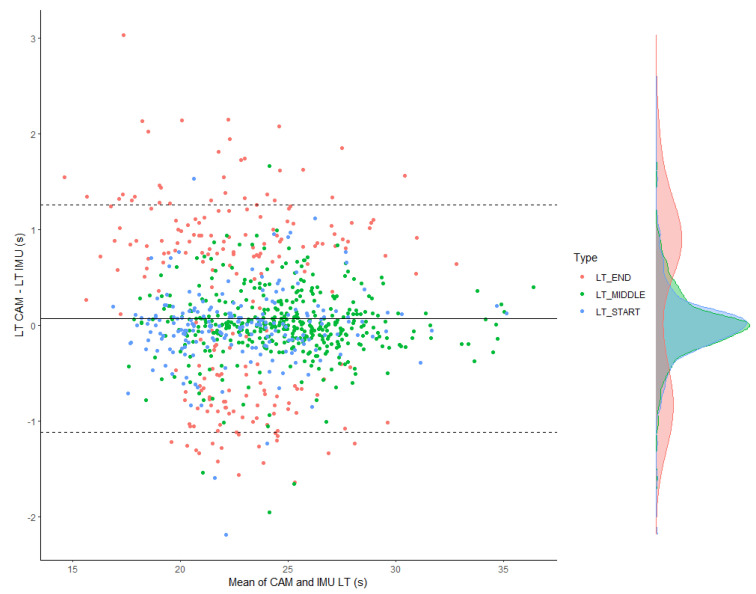
Statistical agreement between gold standard lap times and IMU lap times per type of lap time.

The statistical agreement between LT IMU and LT CAM according to the type of lap is presented in Table 8, and Bland–Altman plots are shown in Figure 4.

The greatest error (systematic ± random) was for LT END, with 0.25 s (0.13; 0.38) ± 0.98 s (0.90; 1.08), whereas the errors are lower and in the same range. Indeed, LT START and LT MIDDLE revealed errors of 0.01s (−0.04; 0.06) ± 0.39 s (0.36; 0.43) and 0.00 s (−0.03; 0.03) ± 0.37 (0.34; 0.39), respectively.

## 4. Discussion

This study aimed to develop a deep learning model devoted to analysis of swimming using a single IMU attached to the sacrum. In particular, the proposed methodology was set up for the purpose of classifying swimming activities at several swimming velocities that may occur during a full training session. A second purpose was to assess the performance of the model in automatically calculating lap times during the exercise.

Previous studies investigated lap detection and/or swimming technique identification using a single IMU sensor located on the sacrum [27], head [50], chest [18], or wrist [16], or in multiple sensor locations [15]. In the present study, the sensor was placed on the sacrum, which is a convenient placement in terms of comfort, safety, and minimal obstruction of movement [20]. Moreover, a recent study has shown that the highest performance in both lap detection and swimming technique identification were achieved with a sensor placed on the sacrum [15].

Most previous papers collected data from relatively homogeneous groups of swimmers. Indeed, the data used for the classification algorithm settings independently considered elite level [20], college level [18], national second league level [16], and national level [15] swimmers. Thus, each of these studies were likely to rely on similar swimming techniques. This homogeneity in terms of the swimming level may affect the process of model training, as the resulting model may fail to generalize the algorithms to swimmers with different skills and levels. In the present study, a strong restriction on swimming level was imposed in the inclusion of participants. As the present database included elite and non-elite swimmers from the regional to the second league level to participation in the national championship, the dataset used here was representative of a large variety of stroke techniques and levels. This suggests that the proposed model can be used reliably with a wide range of swimming proficiency levels. Moreover, many previous studies involved small numbers of participants, i.e., N = 3 [51], N = 11 [52], N = 13 [25], and N = 17 [15]. Apart from [16,18], who collected data from a large number of swimmers, i.e., 45 and 40, respectively, to the best of out knowledge, the data collection presented in this manuscript represents one of the largest databases collected in the literature to date regarding IMU-based swimming activity classification. Moreover, with strong inter- and intra-subject variability, it can be hypothetised that the present database covers most of the swimming skills and techniques needed to train an in-field generalisable classifier. Furthermore, the literature highlights a clear imbalance in terms of the different swimming actions analyzed. Indeed, most previous studies were restricted to specific swimming techniques or phases. While most classification algorithms have focused on the front crawl and backstroke, studies investigating the butterfly and/or breaststroke are more scarce. Moreover, apart from [15], the identification of swimming microphases, including turns and underwater phases, have not previously been tackled. However, the identification of those phases is decisive in analysis over a full training session, or at least during a swimming set. The deep learning model presented in this manuscript is able to distinguish eight classes, including wallpush, underwater, turn, rest, and the four swimming techniques. Consequently, beyond traditional metrics used to analyse swimming performances during training, this model should be able to monitor a swimmer’s performance on non-swimming phases (turn time, underwater time) as well, and could therefore allow new metrics to be derived, for example, underwater distance covered, which is nowadays an important part of the final performance. Another original aspect of the present manuscript lies in the ability to detect rest periods, which is a crucial components of training monitoring. Furthermore, most previously published studies have focused on homogeneous swimming intensities, introducing poor intra-subject variability. Despite high levels of accuracy obtained, this can raise the question of whether the model can be generalized to different swimming velocities. Indeed, Ref. [15] raised the hypothesis that only machine learning methods may be efficient to deal with inter- and intra-swimmer variability in terms of technique. Finally, incorporation of all four swimming techniques combined with different intensities and variability in the level of expertise during the training stage is the starting point to produce a robust model, which is an important contribution of the present manuscript.

To the best of our knowledge, little attention has been paid to deep learning in the context of swimming analysis using IMU data. However, deep learning is a powerful solution to the development of models adapted to a wide panel of users with generalisation performances that can be controlled during model training. Such models are now used for high complexity classification problems such as computer vision. Therefore, such algorithms may be suitable for the complexity of HAR. Recurrent neural networks such as LSTM networks are considered one of the most efficient approaches in learning dynamics from time series [53]. Moreover, this problem of classification involved sequences for which all time steps are available before performing the prediction. Consequently, it is possible for the network to learn dependencies in both directions of the signal. Indeed, bi-LSTM networks were first designed to learn dependencies on the input sequence as-is and on a reversed copy of the input sequence [42]. Furthermore, the problem of classification faced here is a multivariate classification problems with non-linear temporal dynamics as input. For this reason, the model architecture used several bi-LSTM layers. LSTM layers have memory cells that act as an accumulator or a gated leaky neuron [37]. Increasing the depth of the network allows recombining the learned temporal representations from previous layers to increase the level of abstraction with the new representations [54]. This use of bi-LSTM for swimming classification with temporal series as inputs and eight label classes as target outputs is, to the best of the authors’ knowledge, the main contribution of this model to the state of the art. From a more technical perspective, a difficulty was in reaching a good balance between the number of parameters important enough to be used in modeling the whole classification problem without leading to any important variance during the use of unknown data (this variance would be a consequence of overfitting). In order to reach this objective, we tried to make the ratio between parameters and the number of samples input to the model ultimately tend towards 0. In order to fulfill this objective, an architecture with four successive layers of bi-LSTM was chosen, with a decrease in units followed by one dense layers. Moreover, important care was taken to not overfit the data during training. To achieve this, deep learning techniques such as dropout [55] and recurrent dropout [56] were applied in the bi-LSTM layers and dense layers during training. Furthermore, the performance between the training, validation, and testing sets was compared in order to control the tradeoff between bias and variance.

This methodology finally led us to predict swimmers’ activity for each frame recorded during their activity, i.e, every 0.02 s. Recent studies have developed novel approaches to increase the temporal precision of such predictions, such as the one in [20]. More specifically, these authors used a synthetic minority oversampling technique (SMOTE) [57] relevant for microphase analysis. The only paper considering numerous microphases is that of [15], who performed SAR in the same eight classes we investigated, namely, wallpush, underwater, turn, rest, and the four main swimming techniques. However, with a single intensity at 80% of maximal speed, their database suffered from a lack of generalisation; this would require various intensities to be encountered during training. The present manuscript, in contrast, tried to attain high temporal precision with numerous types of activities predicted at very different speeds and intensities.

The literature regarding SAR is composed of three main methodologies: signal processing methods, classical machine learning methods, and deep learning methods. Signal processing methods are the most investigated methodology and have shown good results, with up to 100% stroke recognition and up to 99% the lap segmentation [25]. Regarding machine learning methodologies, they have shown good results, with an accuracy up to 98.63 ± 1.9%, 99.04 ± 0.91%, 99.10 ± 1.43%, and 97.24 ± 1.71% for butterfly stroke, breaststroke, backstroke, and front crawl, respectively [51]. Furthermore, Ref. [58] showed that combining several consecutive predictions led to 100% good predictions. More recently, Ref. [20] reached F1-scores greater than 0.99 with a stroke-by-stroke approach, realizing better temporal precision in the identification of stroke types. Finally, Ref. [16] used a deep learning method to reach an average F1-score of 97.4. In the present manuscript, with F1 scores of 0.93, 0.99, 0.84, and 0.99 for the butterfly, backstroke, breaststroke, and front crawl, respectively, the results are in the same range as the results of most previous studies with respect to stroke recognition.

More generally, the choice presented in this paper, i.e., to make predictions using a sliding window overlapped frame per frame, showed excellent performance over most of the eight classes. Indeed, the weighted averaged overall F1-score after network training and filtering was 0.96, although the F1-score reached by each class showed a high variability between different classes’ precision and recall. Indeed, very short classes such as wallpush and turns were those with the poorest precision, with F1-scores of 0.28 and 0.82, respectively. This poor identification may be due to the duration of phases that are very short, leading to a lower representation in the database of those phases and consequently to fewer opportunities for the model to train on such temporal configurations. However, identifying a wall push is not systematically necessary to identify lap times, because the identification of pre- and post-phases may be sufficient. Moreover, the analysis of the classification report showed that wallpush is most of the time confused with either the underwater, turn, or rest phases, which are the adjacent classes. This analysis suggests that there is not a misclassification of wallpush with other classes; rather, the model has difficulty identifying boundaries between turns, underwater and rest phases, and wall pushes. Moreover, most wall pushes appeared to last less than a few tenths of second. Therefore, using the adjacent classes does not alter the precision of our results. Another tendency that can be pointed out is that of the underwater phase to be misclassified with butterfly and breaststrokes in certain cases. This intra-phase confusion can be explained by the similarity between the sacral kinematics of underwater phases and simultaneous stroke styles that include undulatory movements. However, for classical phase identification the performance is excellent, as previously mentioned. Thus, the model presented in this manuscript may be promising for a wide range of applications. Several key variables in the monitoring of swimming training can be derived from the prediction of swimmers’ activity. Indeed, the time spent underwater and turning time are variables of transition phases that, while poorly investigated by coaches, represent up to one third of the final performance [59]. In addition to the classical variables (mean swimming speed per lap, lap times, etc.), the use of this model may be of great value for elite swimmers and coaches.

However, in order to make further comparisons with the existing literature, it has to be noted that whereas most of the previous studies performed prediction for a session or window with a size ranging from tenths to tens of a second, leading to macro-prediction of swimmers’ activity, this model performs a prediction at every time step, i.e., every 0.02 s. Consequently, it is difficult to compare the performance of this model with the existing literature using predictions with a significant difference in temporal precision. Moreover, several authors adjusted their algorithm to the homogeneity of their population [25], which is convenient when working with a small and identical group but not generalizable when working with wide panels of unknown swimmers. Therefore, another contribution of the present manuscript is the development of a model suited for any level of swimmer and intensity of swimming, regarding the variability embodied in the database. Such precision has, to the best of our knowledge, never been reached in the previous literature aiming to classify swimming activity with machine learning models.

Our results on lap times highlights a minimal loss compared to stopwatch measurement (3.84% vs. 12.12%), demonstrating the relevance for coaches of such an automated method. This may help coaches to automatically monitor many swimmers at the same time. In comparison with previous works based on thresholds, the present results based on a large data base show lower differences between IMU-based and video-based lap times. Indeed, a difference of 0.06 ± 0.6 s was obtained with n = 952 samples, whereas previous works reported differences of 0.72 ± 0.26 s with n = 132 [25] and −0.32 ± 0.58 s with n = 164; Ref. [22] reported a typical error of measurement (TEM) of 0.6 s between their model and the stopwatch. In this study, the TEM with the stopwatch was 0.58 s. This systematic error is nearly identical between our model and an expert coach taking lap times with a stopwatch, reinforcing the confidence a trainer can have in such an approach. To fully investigate this criteria, an excellent statistical indicator seems to be the MAPE. In this paper, the MAPE was 1.15% for LT START, 1% for LT MIDDLE, and 4.07% for LT END. For comparison, Ref. [60] found an MAPE of 3.22% for the TritonWear device (TritonWear, Toronto, ON, Canada) over a 144-bout medley, without distinguishing between bout localization. This paper is therefore a strong contribution towards accurate lap time prediction, which can help in gathering a wide panel of swimming performance data. Gathering data with such precision would be of great interest for coaches, allowing them to better monitor swimmers’ performance in a longitudinal way, as well as for scientists, whose objective would be to model swimming performance across seasons. Moreover, to the best of our knowledge, the separation between LT START, LT MIDDLE, and LT END has never been investigated in the literature with such precisions. However, further improvement in the accuracy of this measurement are very possible. Indeed, whereas good homogeneity between LT START and LT MIDDLE can be highlighted, LT END shows the largest MAPE. This discrepancy with other LT elements can be explained by the transition between a swimming phase and a rest phase, which sometimes may not be easily identified when the swimmer does not actually touch the wall at the end of a training session. This finding is in agreement with other studies that report difficulties in identifying the final touch [22,25,30].

## 5. Limits and Perspectives

The present manuscript is among the first to reach such good performance in the classification of swimming activity with this level of temporal precision and data heterogeneity. However, this work suffers from limitations that may engender interesting perspectives for future studies. The database we used was imbalanced, and certain classes were under-represented compared to other swimming activities (i.e., wallpush and turns), leading to lower performance in predicting these short and under-represented activites. Therefore, it would be interesting to use methodologies dealing with imbalanced datasets, such as SMOTE, in order to improve performance on those classes. Moreover, the present model did not take the diving start and leg kicking phases into account. The inclusion of such phases in the algorithm would be an interesting improvement in view of its usability in daily training routines and monitoring. Finally, the network architecture presented here relies mainly on bi-LSTM. It would be interesting to compare different neural network architectures and their respective performance, as has been carried out in several previous papers. A final interesting point involves the inputs of the neural network. We made the choice to use raw IMU data as the input of the model in order to retain the maximum amount of information and not discretize the data. A hybrid approach using raw IMU data and discretized data in the temporal and frequency domains, respectively, as in traditional machine learning, might be an interesting approach and allow the model to attain better performance.

## 6. Conclusions

This study contributes a deep learning model devoted to the analysis of swimming, using a single IMU attached to the sacrum. The proposed methodology was set up for the purpose of classifying eight swimming activities at four swimming velocities that may occur during a full training session. The proposed methodology took high inter- and intra-swimmer variability into account. LTs were directly computed from the classifier thanks to high temporal precision, and were validated against a video gold standard. This model is a first step towards a powerful training assistant able to analyze swimmers with multiple skill levels according to their needs in the context of in situ training monitoring.

## Figures and Tables

**Table 1:** Phase identification used in video labelling and then for feeding into the algorithms.

Name of the Phase	Definition of the Phase Used for Video Labelling
Wallpush (WP)	From the first frame when the swimmer switches from vertical to horizontal position or from the first frame after TU or RS ends to the last frame when the swimmer’s feet touch the wall
Underwater (UN)	From the first frame after WP ends to the first frame when the hands are dissociated before stroking
Butterfly (BU)	From the first frame after UN ends when the swimmer performs BU style to the first frame when the swimmer’s hands touch the wall
Backstroke (BA)	From the first frame after UN ends when the swimmer performs BA style to the first frame when the swimmer starts the last arm pull before rotating
Breaststroke (BR)	From the first frame after UN ends when the swimmer performs BR style to the first frame when the swimmer’s hand touch the wall
Frontcrawl (FR)	From the first frame after UN ends when the swimmer performs FR technique to the first frame when the swimmer starts the last arm pull before rotating
Turn (TU)	From the first frame after BU, BA, BR, or FR ends to the first frame when the swimmer’s feet touch the wall before leg extension
Rest (RS)	From the first frame after BU or BA ends or first frame when the swimmer’s hand touches the wall during FR or BA, then from rest to the first frame of WP following a rest period

**Table 2:** Network architecture and tuning.

Layer Type	Output Shape	Activation Function	Dropout	Recurrent Dropout	Number of Parameters
Input layer	(64,90,6)				
Bi-LSTM	(64,128,6)	Tanh	0.25	0.25	79,360
Bi-LSTM	(64,64,6)	Tanh	0.25	0.25	41,216
Bi-LSTM	(64,32,6)	Tanh	0.25	0.25	10,368
Bi-LSTM	(64,32)	Tanh	0.25	0.25	6272
Flatten	(64,32)				0
Dense	(64,50)	ReLU	0.5		1650
Batch Normalization	(64,50)		0.5		200
Dense	(64,8)	Softmax			408

**Table 4:** Definition of phases and events to compute IMU lap times.

	START	MIDDLE	END
Frontcrawl and Backstroke	Time difference between the last prediction of a wall push and next-to-last prediction of a wall push or first underwater prediction	Time difference between last wall push prediction or first underwater prediction and the next last turn prediction	Time difference between last wall push prediction or first underwater prediction and next first rest prediction
Butterfly and Breaststroke	Time difference between the last prediction of a wall push and next first prediction of a turn	Time difference between prediction of a turn and the next first prediction of a turn	Time difference between prediction of a turn and the next first rest prediction

**Table 5:** Performance results in swimming activity recognition on testing set before and after filtering step ($\hat{Y}$–${\hat{Y}}_{f}$).

Class	Precision	Recall	F1-Score	n
Wallpush	0.17–0.53	0.08–0.19	0.11–0.28	581
Underwater	0.71–0.92	0.71–0.91	0.71–0.91	3519
Butterfly	0.98–0.99	0.83–0.88	0.90–0.93	7280
Turn	0.58–0.75	0.71–0.91	0.64–0.82	1553
Backstroke	0.96–0.98	0.94–0.99	0.95–0.99	6531
Breaststroke	0.83–0.89	0.95–0.98	0.89–0.94	7117
Frontcrawl	0.96–0.99	0.96–0.99	0.96–0.99	6124
Rest	0.99–0.99	0.99–0.99	0.99–0.99	17,539
Accuracy			0.91–0.96	50,244
Average	0.77–0.88	0.77–0.86	0.77–0.86	50,244
Weighted Average	0.92–0.96	0.91–0.96	0.91–0.96	50,244

**Table 6:** Statistical agreement between lap times computed by IMU and gold standard video (n = 952 laps) respectively.

Mean ± SD LT CAM	Mean ± SD LT IMU	TEM [IC]	Biais [IC]	MAPE ± SD
23.73 ± 3.39 s	23.79 ± 3.35 s	0.60 s [0.57 s; 0.63 s]	0.06 s [−0.05 s; 0.14 s]	1.77 ± 1.82%

**Table 7:** Statistical agreement between lap times computed by stopwatch and gold standard video (n = 870 laps).

Mean ± SD LT CAM	Mean ± SD LT IMU	TEM [IC]	Biais [IC]	MAPE ± SD
23.74 ± 3.39 s	23.63 ± 3.38 s	0.58 s [0.55 s; 0.61 s]	−0.10 s [−0.14 s; −0.07 s]	1.43 ± 2.04%

**Table 8:** Statistical agreement between lap times assessed by IMU and gold standard video according to lap time type.

n	Type	Mean ± SD LT CAM	Mean ± SD LT IMU	TEM [IC]	Biais [IC]	MAPE ± SD
243	START	22.64 ± 3.27 s	22.65 ± 3.20 s	0.39 s [0.36 s; 0.43 s]	0.01 s [−0.04 s; 0.06 s]	1.15 ± 1.31%
470	MIDDLE	24.96 ± 3.18 s	24.96 ± 3.17 s	0.37 s [0.34 s; 0.39 s]	0.00 s [−0.03 s; 0.03 s]	1.00 ± 1.06%
239	END	22.41 ± 3.15 s	22.67 ± 3.06 s	0.98 s [0.90 s; 1.08 s]	0.25 s [0.13 s; 0.38 s]	4.07 ± 1.93%

## Abbreviations

The following abbreviations are used in this manuscript:

IMU	Intertial Measurement Units
STA	Soft Tissue Artefact
HAR	Human Activity Recognition
ML	Machine Learning
RF	Random Forest
SVM	Support Vector Machine
PCA	Principal Component Analysis
DL	Deep Learning
LSTM	Long Short-Term Memory
bi-LSTM	Bidirectional Long Short-Term Memory
CNN	Convolutional Neural Network
RNN	Recurrent Neural Network
BMI	Body Mass Index
WP	Wallpush
UN	Underwater Phase
BU	Butterfly
TU	Turn
BA	Backstroke
BR	Breastroke
FC	Frontcrawl
RS	Rest
LT	Lap Time
MLP	Multilayer Perceptron
LT IMU	Lap Time computed by IMU
LT MAN	Lap Time timed with a Stopwatch
LT CAM	Lap Time timed with a Camera
HOSCV	Hold-Out Subject Cross Validation
TEM	Typical Error of Measurement
MAPE	Mean Absolute Percentage Error
SAR	Swimming Activity Recognition
SMOTE	Synthetic Minority Oversampling Technique
